# Altered IgG *N*-Glycosylation at Onset of Type 1 Diabetes in Children Is Predominantly Driven by Changes in the Fab *N*-Glycans

**DOI:** 10.3390/biomedicines13051206

**Published:** 2025-05-15

**Authors:** Branimir Plavša, Najda Rudman, Flemming Pociot, Olga Gornik

**Affiliations:** 1 Department of Biochemistry and Molecular Biology, University of Zagreb Faculty of Pharmacy and Biochemistry, 10000 Zagreb, Croatia; branimir.plavsa@pharma.unizg.hr (B.P.);; 2 Department of Clinical Research, Steno Diabetes Center Copenhagen, 2730 Herlev, Denmark; 3 Faculty of Health and Medical Sciences, University of Copenhagen, 2200 Copenhagen, Denmark

**Keywords:** *N*-glycosylation, immunoglobulin G, type 1 diabetes, Fc, Fab, IdeS

## Abstract

**Background**: *N*-glycosylation is a post-translational modification involving the attachment of oligosaccharides to proteins and is known to influence immunoglobulin G (IgG) effector functions and even antigen binding. IgG contains an evolutionarily conserved *N*-glycosylation site in its fragment crystallizable (Fc) region, while during V-D-J recombination and somatic hypermutation processes it can also obtain *N*-glycosylation sites in its antigen binding fragment (Fab). Our previous study demonstrated altered IgG *N*-glycosylation in children at type 1 diabetes (T1D) onset, with the most prominent changes involving sialylated glycans, hypothesized to mainly come from the Fab region, however, the analytical method used could not distinguish between Fc and Fab. **Methods**: IgG was isolated from plasma from 118 children with T1D and 98 healthy controls from the Danish Registry of Childhood and Adolescent Diabetes. Isolated IgG was cleaved into Fc and Fab fragments using IdeS enzyme. *N*-glycans were enzymatically released from each fragment, fluorescently labelled with procainamide, and analyzed separately using the UPLC-MS method. Structural annotation of resulting chromatograms was performed using MS/MS. **Results**: T1D related *N*-glycosylation changes were more pronounced in the Fab glycans compared to Fc glycans, with five Fab glycans (Man5, Man7, FA2BG1S1, A2G2S2, FA2BG2S1) being significantly altered compared to only one in the Fc region (FA2[3]BG1). Comparing Fc and Fab glycosylation overall reveals stark differences in the types of glycans on each region, with a more diverse and complex repertoire being present in the Fab region. **Conclusions**: These findings suggest that *N*-glycosylation changes in early onset T1D predominantly originate from the Fab region, underscoring their potential role in modulating (auto)immunity and highlighting distinct glycosylation patterns between Fc and Fab.

## 1. Introduction

Type 1 diabetes (T1D) is a chronic autoimmune disease, characterized by the destruction of the pancreatic islet beta cells, leading to loss of insulin production. The etiology of T1D is not completely understood but is known to involve complex interplay of both genetic and environmental factors [1]. One of the most well-researched factors is the presence of islet-specific autoantibodies, the most common among these being antibodies against glutamic acid decarboxylase (GADA), Zinc-transporter 8 (ZnT8A), tyrosine phosphatase-like protein IA-2 (IA-2A), and insulin antibodies (IA). At disease onset, most patients are positive for multiple autoantibodies, and it was shown that in the prediabetic stage the number of present autoantibodies, rather than the specific autoantibody type, is the most predictive of diabetes risk [2]. Early detection of people at risk of developing T1D is of increasing concern as more effort is being put in delaying or even stopping disease progression [3].

Autoantibodies found in T1D are of class immunoglobulin G (IgG) [4,5], which is a key molecule of the immune system and the second most abundant plasma protein. Its roles range from pathogen opsonization to initiation of destruction of damaged or infected cells through antibody-dependent cellular cytotoxicity (ADCC). Autoreactive IgG is also often involved in development of different autoimmune conditions [6]. IgG has an evolutionarily conserved *N*-glycosylation site present in the fragment-crystallizable (Fc) region, which is present on all IgG molecules. The presence of the *N*-glycans is necessary for binding to Fc receptors [7], and the composition of the glycans influences binding to different Fc receptors [8]. This makes *N*-glycans important modulators of effector functions of IgG such as ADCC and activation of the complement system via both classical and mannose-binding lectin pathway [9]. Besides the Fc glycosylation site, an estimated 15–25% of circulating IgG molecules carry *N*-glycans in the Fab region [10,11]. The diverse repertoire of human immunoglobulin Fab sequences is a result of V-(D)-J recombination as well as somatic hypermutation, which occur during B-cell maturation. Few germline alleles encode for the consensus *N*-glycosylation site Asn-X-Ser/Thr, where X is any amino acid besides proline [11]. Thus the emergence of Fab *N*-glycosylation sites is thought to mainly come during somatic hypermutation [12]. Interestingly, these sites appear to mainly be induced in complementarity determining regions of the variable domain, which suggests it influences antigen affinity. Besides modulating antigen binding, studies have shown that Fab glycans can increase antibody stability or extend its in vivo half-life [13,14]. It can also influence the immunomodulatory effects of intravenous immunoglobulin (IVIG) preparations as research into anti-inflammatory properties of IVIG has shown it is dependent on Fab sialylation [15].

Several methods for analysis of Fab *N*-glycosylation have been described in the literature, bearing different levels of information. The most basic approach separates IgG using sialic acid binding lectin affinity chromatography [16]. This method relies on the fact that most of the *N*-glycans with sialic acid are found in the Fab region of IgG. More sophisticated approaches leverage middle-up analysis using either papain and pepsin or, more recently, IdeS enzymes to cleave IgG into Fab and Fc fractions in such a way that their glycosylation can be analyzed separately [10,17,18,19]. In these studies, after IgG digestion, *N*-glycans were analyzed either using MALDI-TOF or UPLC. Structural analysis using just UPLC relies on exoglycosydase digestion, which is a cumbersome process and is limited by enzyme availability and specificity. MALDI-TOF measurement on the other hand enables a certain level of structural determination, but an orthogonal LC-MS/MS approach gives the highest level of structural information [20].

In a previous study by our research group, *N*-glycosylation of total IgG as well as total plasma protein *N*-glycosylation was shown to be altered in children at onset of T1D when compared to their healthy siblings. Both total plasma and IgG *N*-glycans showed a great discriminative potential [21]. We also showed that the majority of changes found in children with T1D persist to adulthood [22]. The significant changes in IgG glycans were mostly connected to complex sialylated and oligomannose structures, which are known in the literature to mainly come from the Fab region, although they are present on the Fc region as well. Here, we employed a UPLC-MS method, combined with IdeS digestion of IgG to compare *N*-glycosylation of IgG regions separately on a subset of samples from our previous study to determine whether the observed changes originate from Fc or Fab region.

## 2. Materials and Methods

### 2.1. Samples

We analyzed blood plasma samples of subjects with T1D and healthy controls obtained through the Danish Registry of Childhood and Adolescent Diabetes (DanDiabKids) [23]. The cohort was a subpopulation of a larger cohort, which we used to study IgG and plasma protein *N*-glycosylation previously [21]. In total, 216 samples were analyzed, 118 samples of children with newly diagnosed T1D and 98 age- and sex-matched controls without type 1 diabetes but who had a sibling with T1D. The matching procedure ensured that the chosen controls were not siblings of the patients included in our cohort. Population characteristics are shown in Table 1. Overall, samples were evenly matched according to age; however, the T1D group had a slightly higher proportion of female subjects. This was due to being constrained by sample availability.

### 2.2. IgG Isolation from Plasma

IgG isolation was performed using affinity chromatography on a Protein G monolithic plate as described previously [24], the main difference being the use of a Protein G plate with a smaller column volume, which enables more efficient isolation of IgG from lower sample volumes. Briefly, 30 μL of plasma diluted with 210 μL 1xPBS was loaded onto a Protein G plate (50 μL column volume, BIA Sartorius, Ajdovščina, Slovenia). Samples were washed three times with 1xPBS and eluted using 250 μL of 0.1 M formic acid. Eluted IgG was neutralized using 42.5 μL 1 M ammonium bicarbonate and stored at −20 °C until further analysis.

### 2.3. On-Bead Digestion of IgG Using IdeS

IgG was separated into Fc and F(ab)_2_ fragments using IdeS protease (Promega Corporation, Madison, WI, USA) to enable separate analysis of Fc and Fab *N*-glycosylation. A total of 20 μL of IgG Fc capture-select bead suspension (Thermo Fisher Scientific, Waltham, MA, USA) was loaded onto an Oro-flex I filter plate (10 μm polyethylene frit) (Orochem Technologies, Naperville, IL, USA) filter plate and washed four times with 1xPBS. The entire IgG eluate (292.5 μL) was transferred to the filter plate with the Fc beads and allowed to bind for 1 h while gently shaking. The filter plate was then placed on a vacuum manifold and washed four times with 1xPBS. Bound IgG was digested by adding 35 μL of IdeS enzyme in 1xPBS pH 6.6 (1.43 U/μL). The filter plate was sealed and placed in a humid chamber to incubate overnight at 37 °C. The F(ab)_2_ fraction was collected by centrifugation at 50× *g* for 2 min. The Fc fragment bound to the beads was then washed on a vacuum manifold 3 times with 1xPBS and three times with ultrapure water, eluted by adding 100 μL of 0.1 M formic acid and neutralized with 17 μL of 1 M ammonium bicarbonate. Both fractions were dried under vacuum and either used immediately for *N*-glycan release or stored at −20 °C until further analysis.

### 2.4. N-Glycan Release and Procainamide Labelling

Dried Fc and F(ab)_2_ fragments were denatured and solubilized by adding 30 μL of 1.33% SDS and incubating for 10 min at 65 °C. A total of 10 μL of 4% Igepal CA-630 was added to neutralize the SDS. Finally, *N*-glycans were cleaved off the proteins by adding 10 μL of PNGaseF (0.12 U/μL) (Promega Corporation, Madison, WI, USA) and incubating overnight at 37 °C. Released glycans were labelled with procainamide via reductive amination. A total of 25 μL of labelling mixture consisting of 0.96 mg of procainamide hydrochloride and 1.12 mg of 2-methylpiridine–borane complex in 30:70 (*v*/*v*) acetic acid DMSO mixture was added to the sample. The reaction was performed at 65 °C for 2 h. The reaction mixture was diluted with 700 μL of cold acetonitrile (CAN) and transferred to a 0.2 μm SUPOR filter plate (PALL Corporation, New York, NY, USA) for solid phase extraction (SPE). Glycans were washed five times with 96% (*v*/*v*) ACN and finally eluted by adding 120 μL of ultrapure water. Eluted glycans were stored at −20 °C until chromatographic analysis.

### 2.5. UPLC Analysis of Labelled N-Glycans

Chromatographic analysis was performed on a Waters ACQUITY H-class coupled with a fluorescence detector with excitation and emission wavelengths set at 310 nm and 370 nm, respectively. A Waters ACQUITY Premier Glycan BEH Amide (2.1 mm × 100 mm, 1.7 μm, 130 Å) column was used for separation and the column temperature was set to 60 °C. The mobile phase consisted of 100 mM ammonium formate as solvent A and ACN as solvent B, and the gradient was set from 25% A to 38% A over 29 min at 0.4 mL/min flow rate. In total, 12.5 μL of sample was mixed with 37.5 μL ACN and 40 μL of the prepared mixture was injected onto the column. Chromatograms were integrated manually, and the proportion of each glycan peak in the sample was calculated as the percentage of peak area in the total chromatogram area.

### 2.6. LC-MS Determination of Glycan Composition

The UPLC-MS/MS method was used to determine the glycan composition of each chromatographic peak. For LC-MS analysis, 10 μL from 12 random samples was pooled and enriched using porous graphitic carbon (PGC) SPE using a modified protocol described by Jensen et al. [25]. Briefly, 50 μL of PGC suspension in methanol (50 mg/mL) was pipetted onto a C18 ZipTip, creating a PGC column. The solvent was removed by centrifugation, and the column was washed twice with 120 μL of ACN and equilibrated with 120 μL of ultrapure water. A total of 120 μL of pooled purified *N*-glycans was loaded onto the column and washed twice with 60 μL of ultrapure water. Glycans were eluted from the column using 50 μL of 100 mM ammonium formate in 25% can, and 40 μL of the eluate was injected onto the column. A Waters Synapt G2-Si ESI-qTOF mass spectrometer and a Waters ACQUITY H-class UPLC were used for LC-MS analysis. The chromatographic column and method were identical to the UPLC-FLR analysis. MS analysis was performed in positive mode with data-dependent analysis being deployed to obtain MS/MS spectra of 2 most abundant ions in the spectrum. Glycan composition and structure were assigned based on *m*/*z* values as well as fragmentation spectra.

### 2.7. Statistical Analysis

Linear models were used to test for differences in relative areas of *N*-glycans between T1D and control groups. Due to right-skewed distribution of relative areas, data were log-transformed prior to statistical analysis to obtain a normal distribution and enable the use of parametric tests such as linear modelling. Normality was assessed using the Shapiro-Wilk test. The *N*-glycan relative area was treated as the dependent variable with diabetes status as the independent variable. Age and sex were included in the model as covariates. The regression coefficients obtained by linear regression represent the differences in means between the two groups (controls and T1D) but due to log transformed data represented the difference in the logarithm. Transforming the coefficients by exponentiation gives the ratio of the means between the compared groups. *p*-values represent the probability of observing the measured difference if the true difference in the means of the populations was zero, the test is two-tailed. *p*-value was adjusted for multiple testing using the Benjamini–Hochberg method. All statistical analyses and data visualization were performed in R version 4.3.3. A comparison of Fc and Fab glycans’ structural traits in the overall population was conducted using the Wilcoxon rank sum test, as the data are not normally distributed, and we are not interested in the exact magnitude of the difference observed as we were when we compared T1D and control groups.

## 3. Results

### 3.1. Greater Structural Complexity of Fab N-Glycans Compared to Fc

Structural determination of *N*-glycan structures present on Fc and Fab fragments of human IgG was achieved using UPLC-MS/MS. Using a fluorescent detector for quantification offers a high level of reliability and sensitivity and combined with tandem mass spectrometry enables detailed structural characterization with accurate *m*/*z* measurements, fragmentation spectra analysis as well as retention time, informing determination of *N*-glycan structures in each chromatographic peak. HILIC chromatograms of Fc *N*-glycans were separated into 21 glycan peaks (GP) corresponding to 21 unique *N*-glycans structures, while the Fab chromatogram was separated into 25 GPs, containing 29 unique major glycan structures, with 2 structures being a minor part of a peak for a total of 31 unique structures. Overall, Fab *N*-glycans displayed greater structural diversity, making it more difficult to obtain perfect separation between individual structures. Besides higher number of structures present, the structures unique to Fab region exhibited higher level of processing, containing much higher levels of bisecting and sialylated structures.

**Figure 1:**
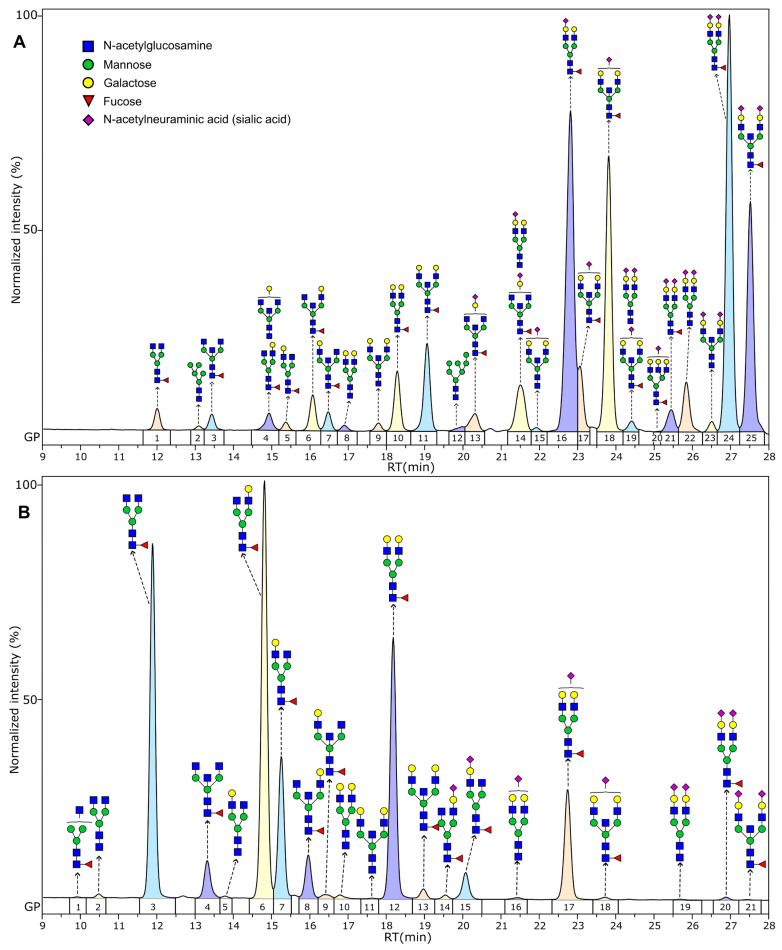
Representative UPLC-FLR chromatograms of Fab (**A**) and Fc (**B**). Major structures in each peak are shown above the respective peak. Peak colors are just for visual differentiation of nearby peaks and bears no information about the peak itself.

The major *N*-glycan structure in each GP and a representative chromatogram of Fc and Fab glycans is shown in Figure 1, while the exact *N*-glycan composition of each GP, as determined by LC-MS, is given in Supplementary Table S1. The level of galactosylation, sialylation, core fucosylation, and bisecting *N*-acetylglucosamine (GlcNAc) was assessed by calculating the relevant structural traits. These traits were obtained by summation of relative abundances of directly measured glycans containing the corresponding structural motif (e.g., sialic acid for the sialylation structural trait). The formulas for their calculation are given in Supplementary Table S2, while the levels of these traits in Fc and Fab in the overall study population are shown in Table 2. The Fab region showed much higher levels of sialylation, with 81% of glycans containing at least one sialic acid residue compared to 14% in the Fc region, as well as a higher proportion of structures with bisecting GlcNAc residues, with 50% compared to 11% on Fc. Oligomannose glycans, specifically those with five (Man5) and seven (Man7) mannose units, while comprising a small percentage of the overall glycans (1%), were detected in the Fab region, and no such glycans were detected in the Fc region. Comparing galactosylation, in the Fc region there is a roughly an even abundance of agalactosylated, monogalactosylated, and digalacotsylated species, while in the Fab region, 86% are digalactosylated. Core fucosylation, which is a distinct feature of IgG *N*-glycosylation, is less pronounced on the Fab region (90% vs. 98%).

### 3.2. IgG N-Glycosylation Changes at T1D Onset Mainly Originate from the Fab Region

In the Fab region of IgG, five *N*-glycan chromatographic peaks, containing six different structures, were significantly increased in children with T1D. This included two oligomannose *N*-glycans, Man5 (GP02) and Man7 (GP12), two biantennary sialylated glycans without core fucose, A2G2S1 (GP14) and A2G2S2 (GP19, GP22), as well as two sialylated bisecting structures FA2BG1S1 (GP14) and FA2BG2S1 (GP19). Bisialylated glycan A2G2S2 was present in two peaks as sialic acid has two possible linkage isomers, α2–6 and α2–3, which are differentially retained on the HILIC column. These isomers cannot be separated based on *m*/*z* values or fragmentation spectra and would require even more complex work-up procedures to distinguish [26,27].

**Figure 2:**
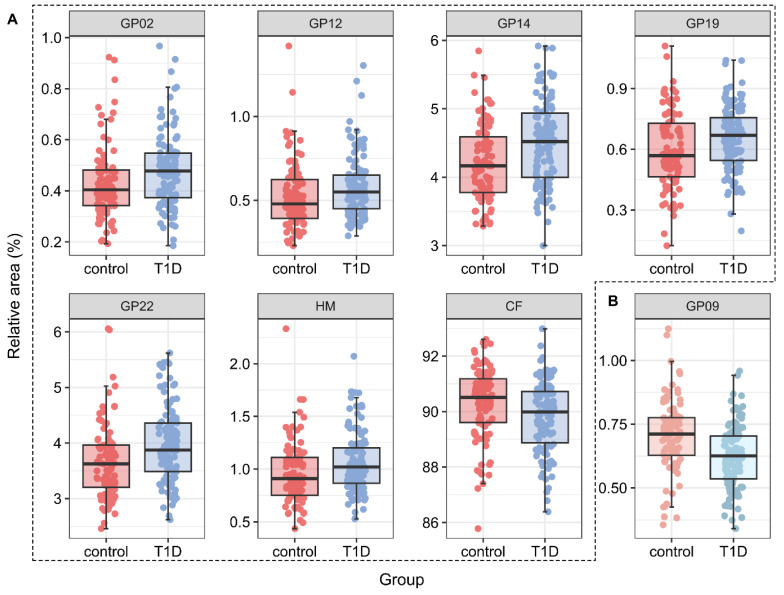
Boxplots of significant changes in Fab glycans (**A**) and Fc (**B**) glycans. The middle line denotes the median, the box is bounded by Q1 and Q3, and whiskers extend to the last data point at most 1.5 times IQR above Q3 or belove Q1. All data points are overlaid above the boxplot, the *y*-axis is true to the values, the *x* axis is randomly dispersed to prevent overplotting (position jitter in R). The magnitude of changes and *p*-values after adjusting for age and sex variance using linear regression are given in Table 3.

When looking at calculated structural traits, oligomannose structures and core-fucosylated structures were increased in children with T1D. In the Fc region of IgG, changes related to T1D were less pronounced than in the Fab region, with only one *N*-glycan being significantly altered (Figure 2). Monogalactosylated glycan with besting GlcNAc and core fucose, FA2[3]BG1 (GP09), was found to be decreased in the T1D group compared to healthy controls (β = 0.885, *p* = 0.0005). Statistically significant differences in both regions are given in Table 3.

All of the Fab glycans significant in this study, apart from Man7, were also significant in our previous study on total IgG in T1D [21]. However, the method used in that study does not detect Man7. The structures Man5, Man7, FA2BG1S1, and FA2BG2S1 are unique to the Fab region. On the other hand, A2G2S1 and A2G2S2 are also present on the Fc region, but they are less abundant there and were not found to be significantly altered in the Fc region in this study, showing that altered Fab *N*-glycosylation was driving the changes observed in our previous study. Interestingly, FA2[3]BG1, a glycan present in both regions, was only found to be significantly changed in the Fc region where it exhibited decreased levels in T1D, an association opposite to the one we previously observed in the total IgG. This could be attributed to the fact that in this study it was more prevalent on Fab (average relative abundances 1.64% and 0.66% on Fab and Fc, respectively) where it showed a slight but statistically insignificant increase (β = 1.046, *p* value = 0.411).

## 4. Discussion

The goal of this study was to further the understanding of IgG *N*-glycosylation changes found in T1D in children at disease onset. Our previous study found that *N*-glycosylation of IgG is significantly altered in T1D, and even showed a good discriminatory potential with a predictive model based on IgG *N*-glycans achieving an AUC of 0.869 [21]. In that study, most of the significant associations were found with sialylated *N*-glycans, which previous research indicates come from the Fab region of IgG [10]. This suggested that the changes were driven by altered Fab *N*-glycosylation. To test this hypothesis, we employed a UPLC-MS method combined with enzymatic digestion by IdeS enzyme to separately measure Fc and Fab *N*-glycans. To our knowledge, this is the first study to utilize LC-MS/MS for structural characterization of IgG Fc and Fab *N*-glycans; thus, this approach to middle-up IgG analysis also brought more information about the differences between Fc and Fab *N*-glycosylation in general.

We have analyzed 216 samples in total, 118 children with T1D and 98 age- and sex-matched controls. The samples included in this study were a subset of the previous study’s samples with a slight difference in the way the controls were selected. In the original DanDiabKids cohort, the control group consisted of healthy siblings of patients with T1D but due to constraints concerning sample availability, this could not be ensured for this study. Instead, we opted to select from the control group subjects, which matched the patients on age and sex as best as possible but were not their relatives, meaning that no patient had a sibling in the control group. This makes the groups more heterogeneous based on both genetic and environmental factors, which could increase the variation in the *N*-glycans, making it more difficult to observe more subtle changes associated with T1D. On the other hand, the more heterogeneous population is more in line with the general population.

As noted in the Section 3, all associations we detected were also significant in the previous study, except for Man7, which is a structure not measured in that study, and FA2[3]BG1, which although also had the opposite association to our study. This discrepancy could be attributed to the fact that the FA2[3]BG1 structure is present on both Fc and Fab, showing the opposite, although statistically insignificant change. The inconsistency between the two studies could also be due to the discussed differences in the cohorts. As for the other differences observed, Man5, Man7, FA2BG1S1, and FA2BG2S1 are structures found exclusively on Fab, while A2G2S1, A2G2S2, and the mentioned FA2[3]BG1 are present on both regions, emphasizing the utility of separate Fc and Fab analysis.

Changes in Fab *N*-glycosylation have been implicated in several autoimmune conditions though they have not been investigated in T1D to date. A study by Koers et al. found that the level of autoantibody Fab glycosylation was significantly increased in five out of eight autoimmune diseases they investigated. The diseases that showed an increase were rheumatoid arthritis (RA), systemic lupus erythematosus, myasthenia gravis, pemphigus vulgaris, and ANCA-associated vasculitis [16]. The authors classify these diseases as chronic autoimmune diseases, while the three diseases that did not show enrichment of Fab *N*-glycans were of an acute type. The study used lectin affinity chromatography to separate Fc and Fab regions and did not investigate individual *N*-glycan profiles, only overall levels of Fab *N*-glycosylation. Bondt et al. investigated Fab *N*-glycosylation in RA and found that although the overall level of IgG Fab glycosylation was higher in patients with RA, the observed changes in IgG *N*-glycosylation, consisting of decreased galactosylation, were driven by differences in the Fc *N*-glycans, as there were no significant differences between Fab glycosylation [18]. This highlights the fact that for autoimmune diseases, when looking at IgG *N*-glycosylation, it is not always enough to just assess total IgG *N*-glycosylation or the overall levels of Fab glycosylation, but valuable information can be obtained from analyzing Fc and Fab glycosylation separately.

Glycosylation changes in the Fc region are responsible for changes in effector functions of IgG, such as promoting ADCC or inhibiting inflammation, and both processes are involved in T1D. However, observed differences in the Fc region were only minor, and most of the changes were in the Fab region, suggesting that there is a systematic shift in Fab glycosylation, which is characterized by an increase in high mannose structures, and structures bearing sialic acids. Sialic acids present on the Fab region have been shown to modulate antigen binding through steric or charge-induced repulsion, competing with the antigen for the binding pocket [28], which could be important for new-onset type 1 diabetes since there is evidence that one of the triggers of this disease could be viral infections [29]. Fab glycans can also influence B cell receptor signaling and activation of autoreactive B cells [28].

The advantage of using UPLC-MS/MS is that it enables us to distinguish between structural isomers by analyzing fragmentation spectra. This is especially important for distinguishing between triantennary *N*-glycans and glycans with bisecting GlcNAc, which have the same *m*/*z* values. We found that the Fab region contained exclusively biantennary and bisecting glycans, with only a single minor peak, GP20, being a triantennary glycan, accounting for 0.06% of total structures. This differentiates IgG from other plasma proteins, even other immunoglobulin isoforms, like IgA and IgM, which do contain triantennary glycans both in Fc and Fab regions [30,31].

There is relatively little data about the Fc and Fab *N*-glycosylation in the general population besides what can be inferred from a small number of studies in autoimmune diseases or pooled IVIG preparations. Thus, the exact distribution of Fc or Fab glycans to expect is not known, though a general pattern is observable. In a 2014 study by Bondt et al. Fc and Fab *N*-glycosylation of healthy women during pregnancy and post-partum was assessed using MALDI-TOF analysis after IdeS digestion of IgG. Besides the reported changes during pregnancy, the 26–52 week post-partum samples also provided insight into overall differences between Fc and Fab glycans in a healthy population [17]. The structural features of *N*-glycans differed significantly between the two regions. Comparing the averages to our study we find similar results, with slight differences, which can easily be attributed to the slightly different methods employed, and more importantly, vastly different cohorts, with their study consisting of solely of women, while our study included children of both sexes. Galactosylation was nearly identical, with 94% and 67% for Fab and Fc glycans respectively, compared to our results of 97% and 75%. The slightly higher proportion of galactosylated species in the Fc region is possibly due to the age difference, as it is known to decrease with age in the adult population, although this link is less well established in the younger population due to insufficient research [24]. Levels of neutral, non-sialylated glycans were lower in our study where they were 86% and 17% for Fc and Fab, compared to 81% and 7% percent in their study. One possible explanation for this discrepancy could be because the acidic labelling reaction conditions used for UPLC-FLR analysis could lead to loss of sialic acids, which is not the case in the MALDI-TOF approach. However, the loss of sialic acids is minimal in IgG, as it contains at most two sialic acid residues, and the effect is reported to be more pronounced in complex tri- and tetra-sialylated *N*-glycans [32]. Another possibility is biological, with the study showing that Fab sialylation was markedly higher during pregnancy compared to 26 weeks post-partum; thus, the levels reported by the study could still be elevated compared to the general population. Levels of bisecting structures and high mannose structures were rather similar.

## 5. Conclusions

Our approach to Fc and Fab *N*-glycan analysis gives greater insight into structural diversity of *N*-glycans present on each region as it is the first such study to couple UPLC with tandem MS. This gives a larger amount of structural information compared to previous MALDI-TOF or exoglycosydase approaches; for example, it enables us to distinguish between structural isomers like triantennary glycans and bisecting glycans, which have the same *m*/*z* values. We observe that while Fab *N*-glycans show greater diversity and higher levels of sialylated and bisecting glycans, their repertoire is still limited to only biantennary glycans characteristic for IgG.

Further insight is also provided into the IgG *N*-glycosylation changes in T1D. We have shown that they predominantly originate from the Fab region and although the role of Fab *N*-glycans is less well researched than that of the Fc glycans it is increasingly being noted as a characteristic of autoimmunity. Furthering understanding of *N*-glycosylation of IgG in the development of T1D is important considering that the pathogenesis of T1D is not completely understood, and many questions are still open particularly with regards to disease onset.

## Figures and Tables

**Table 1:** Population characteristics of analyzed samples.

Characteristic		T1D (*n* = 118)	Control (*n* = 98)	*p*-Value ^1^
Age (years) ^2^		11.0 (8.0, 15.0)	11.0 (8.0, 14.0)	0.8
Sex ^3^	F	67 (57%)	50 (51%)	0.4
	M	51 (43%)	48 (49%)	

^1^ Wilcoxon rank sum test; Pearson’s chi-squared test; ^2^ Median (Q1, Q3); ^3^ *n* (%).

**Table 2:** Distribution of calculated structural traits between Fc and Fab *N*-glycans. G0—structures without galactose, G1—monogalactosylated structures, G2—digalactosylated structures, S0—neutral glycans, no terminating sialic acids, S1—monosialylated structures, S2—disialylated structures, B—structures with bisecting GlcNAc, HM—high mannose structures, CF—structures with core fucose.

Structural Trait	Fc ^1^	Fab ^1^	*p*-Value ^2^
G0	26.71 (6.26)	2.75 (1.34)	<0.001
G1	40.56 (2.63)	9.71 (1.99)	<0.001
G2	32.74 (6.07)	86.46 (3.27)	<0.001
S0	86 (2.81)	17.75 (3.28)	<0.001
S1	13.57 (2.7)	42.92 (1.78)	<0.001
S2	0.43 (0.15)	38.32 (3.87)	<0.001
B	10.79 (2.09)	49.86 (5.91)	<0.001
HM	0 (0)	1.01 (0.29)	<0.001
CF	98.02 (0.72)	90.04 (1.29)	<0.001

^1^ Mean (SD); ^2^ Wilcoxon rank sum test.

**Table 3:** Associations of directly measured Fc and Fab glycans and structural traits with T1D, adjusted for age and sex and corrected for multiple testing. Only significant associations are shown; the rest of the data are presented in Supplementary Tables S3 and S4.

Glycan	Description ^1^	Beta Coefficient ^2^	95% CI	*p*-Value	Adjusted *p*-Value ^3^
Fab *N*-glycans					
GP02	Man5	1.119	1.032, 1.214	0.0068	0.033
GP12	Man7	1.127	1.035, 1.226	0.0058	0.033
GP14	FA2BG1S1, A2G2S1	1.070	1.033, 1.109	0.0002	0.005
GP19	A2G2S2, FA2BG2S1	1.132	1.04, 1.231	0.0043	0.033
GP22	A2G2S2	1.090	1.041, 1.141	0.0003	0.005
HM	Oligomannose	1.122	1.043, 1.208	0.0023	0.026
CF	Core fucose	0.995	0.991, 0.998	0.0057	0.033
Fc *N*-glycans					
GP09	FA2[3]BG1	0.885	0.838, 0.935	0.00002	0.0005

^1^ Structure abbreviations: all *N*-glycans have two core GlcNAcs; F at the start of the abbreviation indicates a core fucose α1,6-linked to the inner GlcNAc; Mx indicates the number of mannose residues on core GlcNAcs; Ax indicates the number of antenna (GlcNAc) on the trimannosyl core: A2, biantennary with both GlcNAcs β1,2-linked; B indicates bisecting GlcNAc linked β1,4 to β1,3-mannose; Gx indicates the number of β1,4-linked galactose residues on the antenna; Sx indicates the number of sialic acids linked to galactose. ^2^ Ratio between mean glycan level in T1D and control; ^3^ adjusted using Benjamini–Hochberg.

## Data Availability

The data presented in this study are available on request from the corresponding author.

## Supplementary Materials

The following supporting information can be downloaded at: https://www.mdpi.com/article/10.3390/biomedicines13051206/s1, Figure S1: Boxplots depicting Fab *N*-glycan levels in children with T1D and healthy controls; Figure S2: Boxplots depicting Fc *N*-glycan levels in children with T1D and healthy controls; Figure S3: Boxplots depicting levels of Fab structural traits in children with T1D and healthy controls; Figure S4: Boxplots depicting levels of Fc structural traits in children with T1D and healthy controls; Table S1: *N*-glycan composition of each chromatographic peak in Fc and Fab HILIC chromatograms as assessed by UPLC-MS/MS; Table S2: Formulas for the calculation of derived traits for Fc and Fab glycans; Table S3: Distribution of directly measured Fc *N*-glycans and structural traits in the overall population and in each group and their association with T1D, adjusted for age and sex; Table S4: Distribution of directly measured Fab *N*-glycans and structural traits in the overall population and in each group and their association with T1D, adjusted for age and sex.
