# Supplementary material for: Altered IgG N-Glycosylation at Onset of Type 1 Diabetes in Children Is Predominantly Driven by Changes in the Fab N-Glycans
Source: Biomedicines. 2025 May 15;13(5):1206. doi: 10.3390/biomedicines13051206 (PMC12108837; doi:10.3390/biomedicines13051206)
Supplement: Supplementary file 1 [file biomedicines-13-01206-s001.zip › Supplementary_material_T1D_FcFab.pdf]

Supplementary Materials

Table S2. Formulas for the calculation of derived traits for Fc and Fab glycans. G and S denote galactose and sialic acid respectively, the number represents the number of residues. B – bisecting, HM – high mannose, CF – core fucose.

**Fc glycans**	
G0	GP01+GP02+GP03+GP04
G1	GP05+GP06+GP07+GP08+GP09+GP14+GP15
G2	GP10+GP11+GP12+GP13+GP16+GP17+GP18+GP19+GP20+GP21
S0	GP01+GP02+GP03+GP04+GP05+GP06+GP07+GP08+GP09+GP10+GP11+GP12+GP13
S1	GP14+GP15+GP16+GP17+GP18
S2	GP19+GP20+GP21
B	GP04+GP08+GP09+GP11+GP13+GP18+GP21
HM	*NA*
CF	GP01+GP03+GP04+GP06+GP07+GP08+GP09+GP12+GP13+GP14+GP15+GP17+GP20+GP21
**Fab glycans**	
G0	GP01+GP03
G1	GP04+GP05+GP06+GP07+GP13+1/2*GP14
G2	GP08+GP09+GP10+GP11+1/2*GP14+GP15+GP16+GP17+GP18+GP19+GP21+GP22+GP23+GP24+GP25
S0	GP01+GP03+GP04+GP05+GP06+GP07+GP08+GP09+GP10+GP11
S1	GP13+GP14+GP15+GP16+GP17+GP18+1/2*GP19+GP20
S2	1/2*GP19+GP21+GP22+GP23+GP24+GP25
B	GP03+1/2*GP04+GP06+GP07+GP09+GP11+GP13+1/2*GP14+GP15+GP17+GP18+1/2*GP19+GP23+GP25
HM	GP02+GP12
CF	GP01+GP03+1/2*GP04+GP05+GP06+GP07+GP10+GP11+GP13+1/2*GP14+GP16+GP17+GP18+1/2*GP19+GP20+GP21+GP24+GP25

Table S3. Distribution of directly measured Fc N-glycans and structural traits in the overall population and in each group and their association with T1D, adjusted for age and sex.

Glycan	Description	Overall[1]	control[1]	T1D[1]	Beta coefficient[2]	95% CI	Adjusted *p*-value[3]
GP01	FA1	0.09 (0.03)	0.09 (0.03)	0.09 (0.03)	1.06	-0.025, 0.143	0.3561
GP02	A2	0.27 (0.17)	0.26 (0.15)	0.27 (0.19)	1.033	-0.122, 0.187	0.7557
GP03	FA2	22.1 (5.4)	21.3 (4.8)	22.8 (5.7)	1.061	0, 0.119	0.2948
GP04	FA2B	4.22 (1.24)	4.21 (1.09)	4.23 (1.35)	0.993	-0.078, 0.064	0.8910
GP05	A2G1	0.21 (0.12)	0.22 (0.12)	0.21 (0.11)	0.941	-0.191, 0.07	0.4645
GP06	FA2[6]G1	23.49 (2.07)	23.53 (2.09)	23.46 (2.05)	0.998	-0.026, 0.022	0.8910
GP07	FA2[3]G1	9.33 (1.35)	9.41 (1.34)	9.27 (1.36)	0.988	-0.049, 0.024	0.6058
GP08	FA2[6]BG1	4.15 (0.95)	4.23 (1.00)	4.08 (0.90)	0.966	-0.09, 0.02	0.3561
**GP09**	**FA2[3]BG1**	**0.66 (0.14)**	**0.71 (0.14)**	**0.62 (0.12)**	**0.885**	**-0.176, -0.067**	**0.0005**
GP10	A2G2	0.61 (0.33)	0.64 (0.36)	0.58 (0.30)	0.918	-0.2, 0.028	0.3561
GP11	A2BG2	0.15 (0.06)	0.16 (0.06)	0.14 (0.04)	0.887	-0.219, -0.021	0.1966
GP12	FA2G2	19.5 (3.6)	19.7 (3.1)	19.3 (3.9)	0.975	-0.075, 0.025	0.4393
GP13	FA2BG2	1.22 (0.38)	1.25 (0.38)	1.19 (0.38)	0.952	-0.121, 0.022	0.3561
GP14	FA2[6]G1S1	0.32 (0.06)	0.33 (0.06)	0.32 (0.06)	0.971	-0.078, 0.02	0.3756
GP15	FA2[3]G1S1	2.39 (0.39)	2.45 (0.38)	2.34 (0.40)	0.951	-0.094, -0.006	0.1966
GP16	A2G2S1	0.34 (0.13)	0.35 (0.15)	0.33 (0.11)	0.962	-0.123, 0.046	0.4645
GP17	FA2G2S1	10.18 (2.40)	10.38 (2.29)	10.02 (2.50)	0.956	-0.109, 0.018	0.3561
GP18	FA2BG2S1	0.33 (0.08)	0.34 (0.09)	0.32 (0.07)	0.935	-0.126, -0.008	0.1966
GP19	A2G2S2	0.072 (0.019)	0.072 (0.019)	0.073 (0.018)	1.004	-0.062, 0.07	0.9079
GP20	FA2G2S2	0.29 (0.11)	0.29 (0.11)	0.28 (0.10)	0.945	-0.143, 0.03	0.3561
GP21	FA2BG2S2	0.069 (0.045)	0.072 (0.055)	0.067 (0.035)	0.969	-0.141, 0.078	0.6593
G0		26.7 (6.3)	25.9 (5.5)	27.4 (6.8)	1.049	-0.01, 0.105	0.3561
G1		40.56 (2.63)	40.87 (2.50)	40.29 (2.71)	0.986	-0.031, 0.004	0.3561
G2		32.7 (6.1)	33.2 (5.6)	32.3 (6.5)	0.966	-0.085, 0.017	0.3561
S0		86.00 (2.81)	85.71 (2.69)	86.26 (2.89)	1.007	-0.002, 0.015	0.3561
S1		13.57 (2.70)	13.85 (2.58)	13.32 (2.78)	0.955	-0.099, 0.008	0.3561
S2		0.43 (0.15)	0.44 (0.16)	0.42 (0.14)	0.957	-0.123, 0.035	0.4011
B		10.79 (2.09)	10.97 (2.15)	10.64 (2.04)	0.97	-0.08, 0.019	0.3714
CF		98.02 (0.72)	97.95 (0.78)	98.07 (0.67)	1.001	-0.001, 0.003	0.3561

[1]Relative area (%), mean (SD)

[2]Ratio between mean glycan level in T1D and control, adjusted for age and sex

[3]Adjusted using Benjamini-Hochberg

Table S4. Distribution of directly measured Fab N-glycans and structural traits in the overall population and in each group and their association with T1D, adjusted for age and sex.

Glycan	Description	Overall[1]	control[1]	T1D[1]	Beta coefficient[2]	95% CI	Adjusted *p*-value[3]
GP01	FA2	1.41 (0.83)	1.46 (0.89)	1.38 (0.77)	0.992	-0.165, 0.15	0.924
**GP02**	**M5**	**0.46 (0.14)**	**0.43 (0.14)**	**0.48 (0.14)**	**1.119**	**0.031, 0.194**	**0.033**
GP03	FA2B	1.34 (0.73)	1.31 (0.69)	1.36 (0.76)	1.021	-0.101, 0.143	0.879
GP04	A2BG1, FA2[6]G1	1.47 (0.59)	1.53 (0.67)	1.42 (0.52)	0.949	-0.162, 0.057	0.614
GP05	FA2[3]G1	0.53 (0.28)	0.55 (0.31)	0.51 (0.26)	0.974	-0.175, 0.122	0.879
GP06	FA2[6]BG1	2.20 (0.67)	2.19 (0.64)	2.20 (0.70)	0.991	-0.084, 0.066	0.892
GP07	FA2[3]BG1	1.64 (0.45)	1.60 (0.44)	1.68 (0.45)	1.046	-0.023, 0.113	0.411
GP08	A2G2	0.42 (0.11)	0.40 (0.10)	0.43 (0.12)	1.071	0, 0.137	0.195
GP09	A2BG2	0.56 (0.17)	0.57 (0.17)	0.56 (0.16)	0.976	-0.096, 0.046	0.700
GP10	FA2G2	3.03 (0.68)	3.07 (0.69)	3.00 (0.67)	0.977	-0.083, 0.037	0.666
GP11	FA2BG2	5.16 (1.04)	5.27 (1.07)	5.06 (1.00)	0.964	-0.086, 0.012	0.372
**GP12**	**M7**	**0.55 (0.19)**	**0.52 (0.19)**	**0.58 (0.18)**	**1.127**	**0.035, 0.204**	**0.033**
GP13	FA2BG1S1	1.69 (0.44)	1.68 (0.40)	1.71 (0.46)	1.004	-0.06, 0.069	0.919
**GP14**	**FA2BG1S1, A2G2S1**	**4.37 (0.61)**	**4.20 (0.56)**	**4.50 (0.61)**	**1.07**	**0.032, 0.104**	**0.005**
GP15	A2BG2S1	0.27 (0.08)	0.27 (0.09)	0.26 (0.07)	0.979	-0.091, 0.049	0.749
GP16	FA2G2S1	17.07 (2.53)	17.02 (2.64)	17.11 (2.45)	1.009	-0.031, 0.049	0.856
GP17	FA2BG2S1	3.74 (0.64)	3.79 (0.68)	3.69 (0.61)	0.976	-0.071, 0.023	0.614
GP18	FA2BG2S1	15.41 (2.42)	15.72 (2.38)	15.15 (2.43)	0.964	-0.074, 0.001	0.199
**GP19**	**A2G2S2, FA2BG2S1**	**0.63 (0.18)**	**0.59 (0.19)**	**0.66 (0.16)**	**1.132**	**0.039, 0.208**	**0.033**
GP20	FA2BG3S1	0.06 (0.03)	0.07 (0.03)	0.06 (0.03)	0.887	-0.263, 0.024	0.291
GP21	FA2G2S2	1.70 (0.42)	1.64 (0.42)	1.75 (0.42)	1.086	0.004, 0.161	0.170
**GP22**	**A2G2S2**	**3.83 (0.68)**	**3.66 (0.66)**	**3.98 (0.67)**	**1.09**	**0.04, 0.132**	**0.005**
GP23	A2BG2S2	0.63 (0.25)	0.67 (0.31)	0.60 (0.19)	0.928	-0.164, 0.014	0.291
GP24	FA2G2S2	17.9 (3.5)	17.6 (3.4)	18.1 (3.5)	1.027	-0.027, 0.079	0.614
GP25	FA2BG2S2	13.94 (2.26)	14.15 (2.30)	13.76 (2.21)	0.971	-0.074, 0.014	0.407
G0		2.75 (1.34)	2.77 (1.33)	2.73 (1.36)	0.991	-0.129, 0.111	0.919
G1		9.71 (1.99)	9.65 (1.94)	9.76 (2.03)	1.008	-0.045, 0.061	0.879
G2		86.5 (3.3)	86.6 (3.2)	86.4 (3.3)	0.998	-0.012, 0.009	0.879
S0		17.8 (3.3)	17.9 (3.4)	17.6 (3.2)	0.981	-0.069, 0.03	0.666
S1		42.92 (1.78)	43.05 (1.70)	42.82 (1.85)	0.996	-0.015, 0.006	0.652
S2		38.3 (3.9)	38.1 (3.8)	38.5 (3.9)	1.012	-0.015, 0.039	0.652
B		49.9 (5.9)	50.4 (5.9)	49.4 (5.9)	0.978	-0.052, 0.008	0.375
**HM**		**1.01 (0.29)**	**0.95 (0.29)**	**1.06 (0.28)**	**1.122**	**0.042, 0.189**	**0.026**
**CF**		**90.05 (1.29)**	**90.32 (1.29)**	**89.82 (1.26)**	**0.995**	**-0.009, -0.002**	**0.033**

[1]Relative area (%), mean (SD)

[2]Ratio between mean glycan level in T1D and control

[3]Adjusted using Benjamini-Hochberg

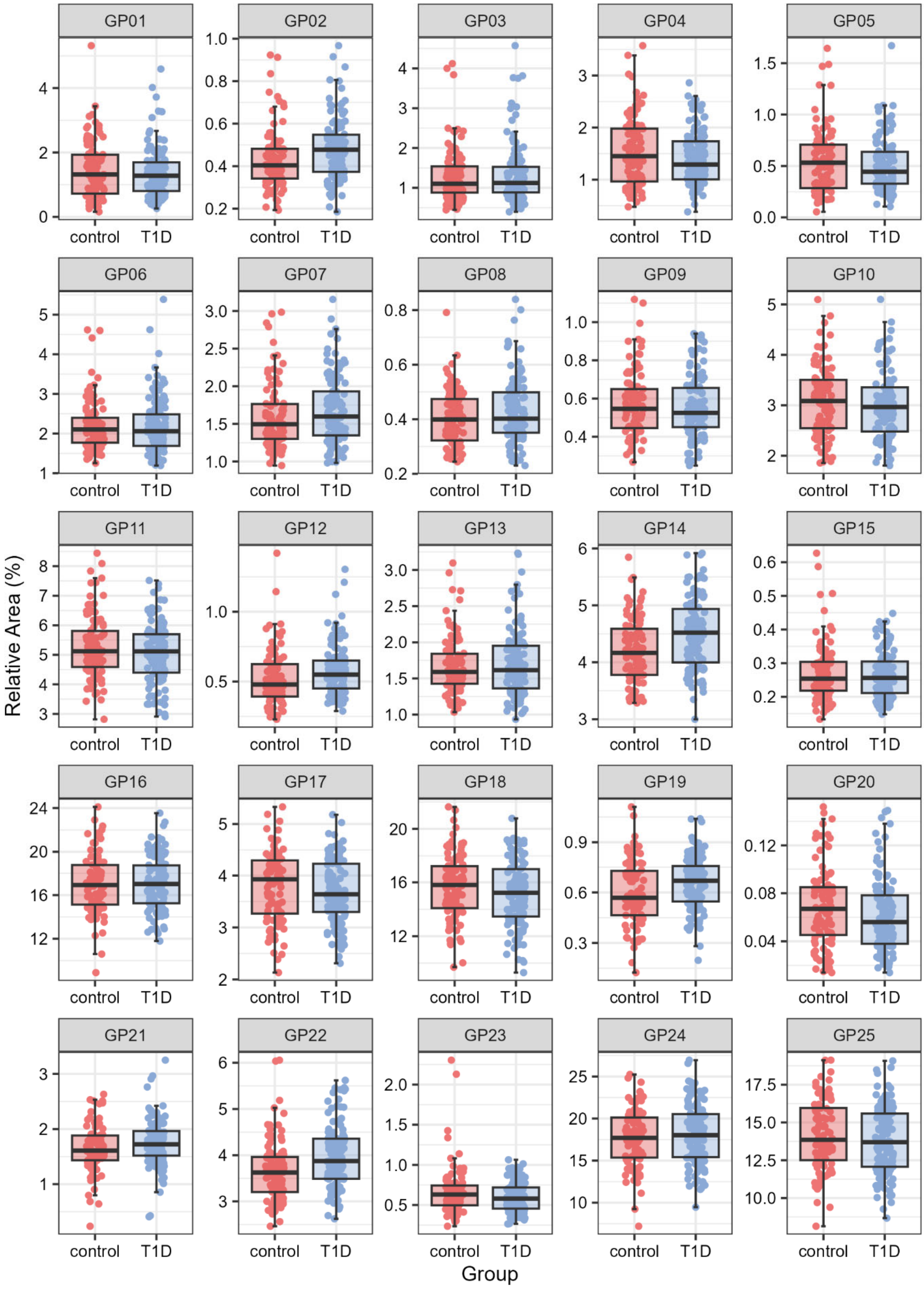

Figure S1. Boxplots depicting Fab N-glycan levels in children with T1D and healthy controls

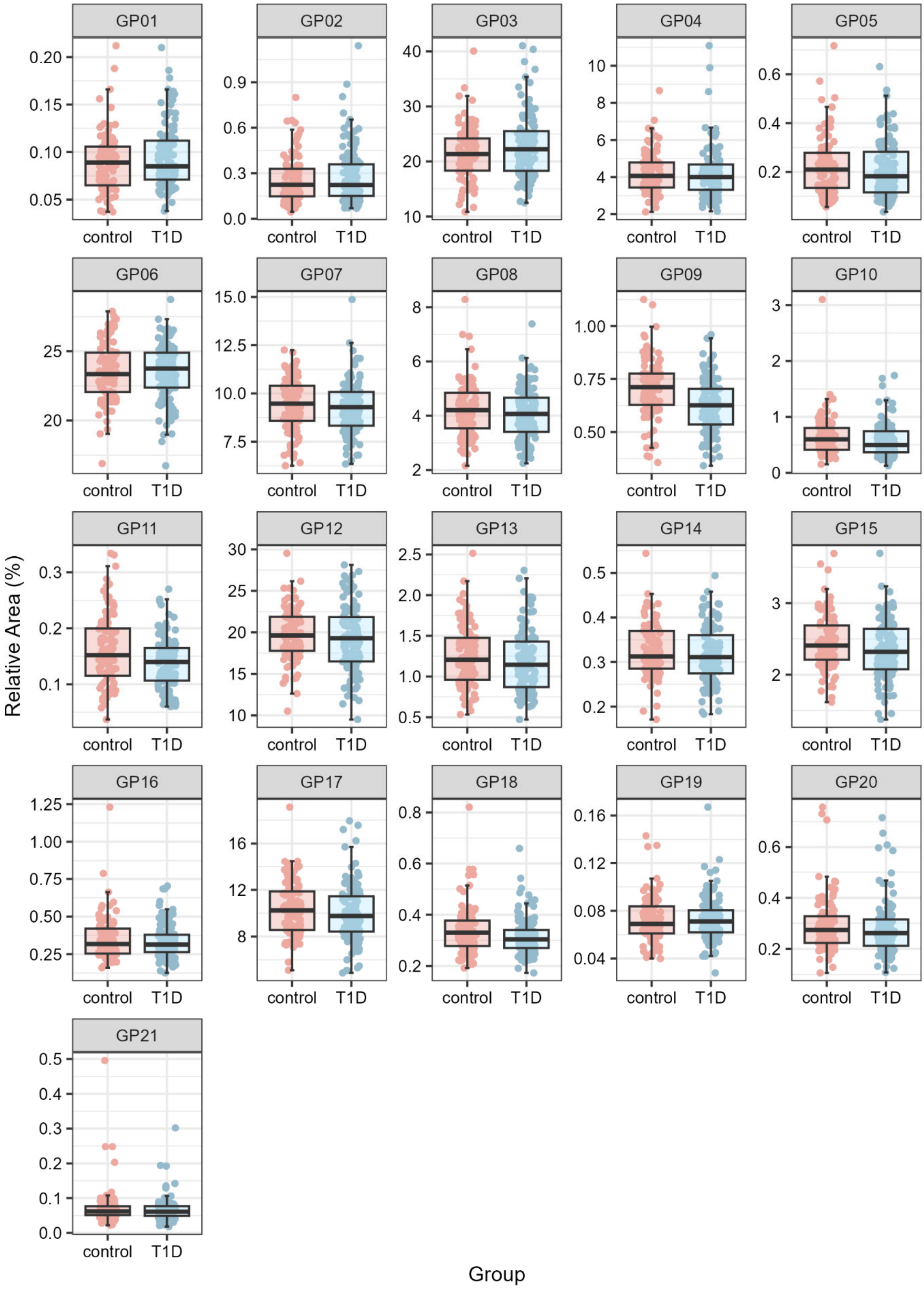

Figure S2. Boxplots depicting Fc N-glycan levels in children with T1D and healthy controls

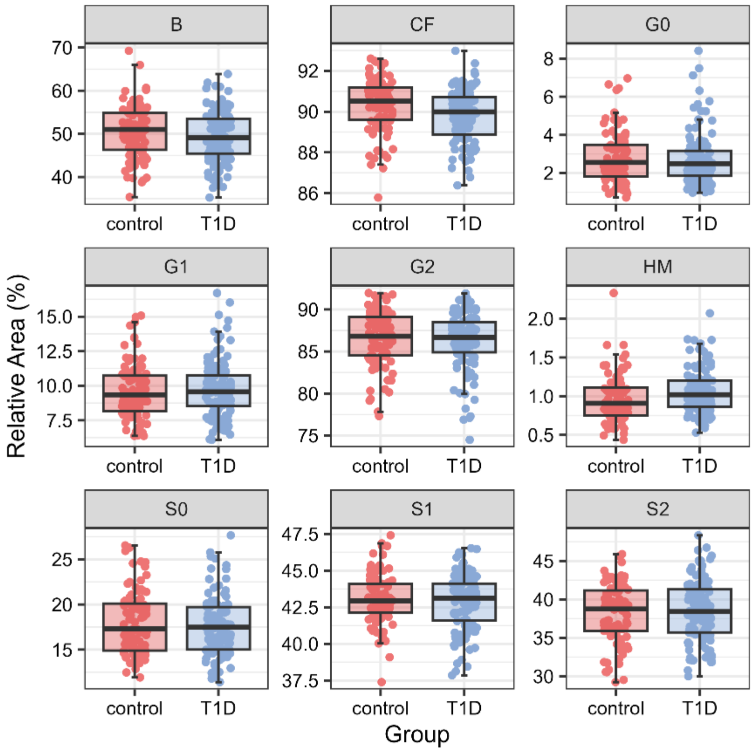

Figure S3. Boxplots depicting levels of Fab structural traits in children with T1D andhealthy controls

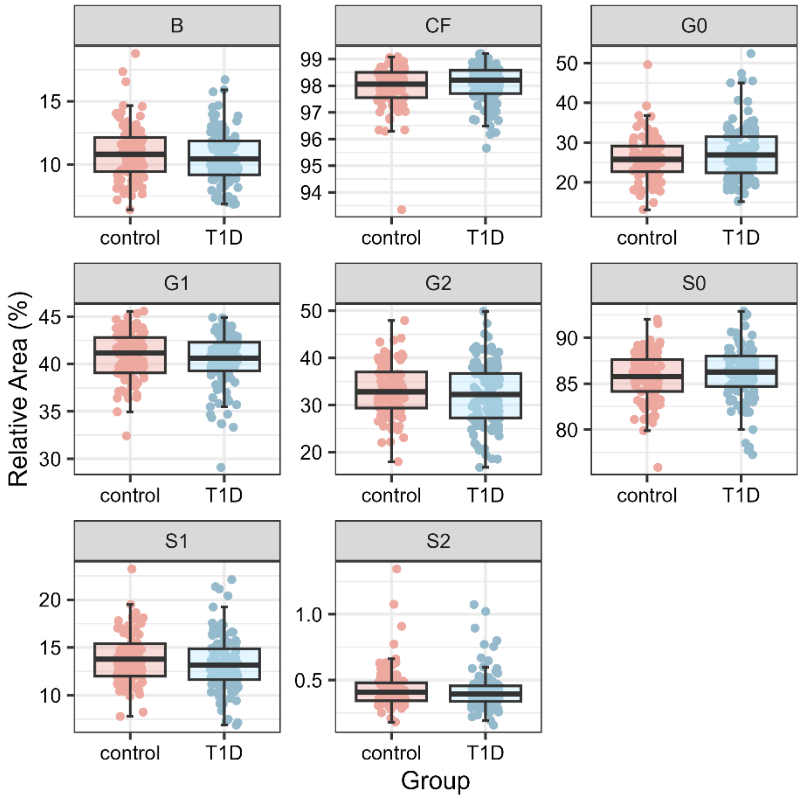

Figure S4. Boxplots depicting levels of Fc structural traits in children with T1D and healthy controls
